# How might infant and paediatric immune responses influence malaria vaccine efficacy?

**DOI:** 10.1111/j.1365-3024.2009.01137.x

**Published:** 2009-09

**Authors:** A M MOORMANN

**Affiliations:** Case Western Reserve University, Center for Global Health and DiseasesCleveland, OH, USA

**Keywords:** infant immunity, malaria, vaccines

## Abstract

Naturally acquired immunity to malaria requires repeat infections yet does not engender sterile immunity or long-lasting protective immunologic memory. This renders infants and young children the most susceptible to malaria-induced morbidity and mortality, and the ultimate target for a malaria vaccine. The prevailing paradigm is that infants initially garner protection due to transplacentally transferred anti-malarial antibodies and other intrinsic factors such as foetal haemoglobin. As these wane infants have an insufficient immune repertoire to prevent genetically diverse *Plasmodium* infections and an inability to control malaria-induced immunopathology. This Review discusses humoral, cell-mediated and innate immune responses to malaria and how each contributes to protection – focusing on how deficiencies in infant and paediatric immune responses might influence malaria vaccine efficacy in this population. In addition, burgeoning evidence suggests a role for inhibitory receptors that limit immunopathology and guide the development of long-lived immunity. Precisely how age or malaria infections influence the function of these regulators is unknown. Therefore the possibility that infants may not have the immune-dexterity to balance effective parasite clearance with timely immune-regulation leading to protective immunologic memory is considered. And thus, malaria vaccines tested in adults and older children may not be predictive for trials conducted in infants.

## Introduction

We often reduce, into a single sentence, the accumulation of decade’s worth of clinical and epidemiologic studies on malaria with an estimated number of cases and of children each year who die. We all agree that events leading to the manifestations of severe malaria in a paediatric population are multi-factorial and are influenced by intrinsic as well as extrinsic factors. These include, but are not limited to, malaria transmission intensity, parasite genetic diversity and complexity of infection, the degree of maternal antibody protection and prenatal malaria experience, foetal haemoglobin, haemoglobin S heterozygosity, and nutritional status. These factors aside, children remain more susceptible to malaria compared to adults. This age-associated susceptibility has in part been explained by children being immunologically naïve whereby repeat infections are required to develop a repertoire of immune cells capable of recognizing the various and antigenically variant malaria-derived proteins. Once infected, however, children are more likely to succumb to the immunopathology associated with malaria [reviewed in (1)] which is primarily the result of insufficient modulation of TNF-α mediated pro-inflammatory responses induced by parasite molecules and antigens that stimulate innate and adaptive immunity [reviewed in (2,3)]. Compounding the complexity of the host–parasite interaction is the fact that natural malaria infections do not confer long-lasting protective (i.e. sterile) immunity. This is illustrated by repeat though less severe infections present in adults and the waning of immunity after leaving a malaria endemic area – hence the terms ‘semi-immune’ or ‘partial-protection’ are used when referring to immunity developed under natural exposure conditions. How the malaria parasite prevents the acquisition of immunologic memory remains unresolved; however, possible mechanisms will be discussed in this review.

To better understand age-associated differences in anti-malarial immunity, immuno-epidemiologic studies of naturally exposed populations have been conducted with the express purpose of discovering which immune mediators may be responsible for semi-protective immunity that could be enhanced or sustained with a malaria vaccine. What immune responses do asymptomatic malaria-infected people possess compared to those who have symptomatic infections? And by extension, which immune responses confer the semi-immune status benefiting adults that are lacking in infants and young children? This review highlights humoral, cell-mediated and innate immune responses to malaria under natural exposure conditions that may contribute to the development of protection – focusing on how ‘deficiencies’ in infant and paediatric immune responses might influence malaria vaccine efficacy in this population. Within this context, malaria vaccines that have shown promise in children will be discussed. As this review concludes, a better understanding of infant immunity is needed in order to design an efficacious malaria vaccine that accomplishes what natural infections do not.

## Humoral Immunity

The most compelling evidence that antibodies are important for protection against malaria arose from experiments in the 1960s where passive transfer of sera from semi-immune adults was used to treat children with clinical *Plasmodium falciparum* (Pf) malaria (4,5). Several recent reviews have summarized what we know about the age- and exposure-dependent development of naturally acquired humoral immunity to multiple malaria antigens that subsequently have been selected as vaccine candidates (6–9). Antibodies against blood stage malaria proteins are viewed as the primary mediators that prevent infection – based on the principle that if antibodies prevent merozoites from invading red blood cells there will be no ensuing pathology. However, cross-sectional sero-epidemiologic studies to identify precisely which anti-malarial antibodies mediate resistance to infection have led to conflicting results and have failed to reveal easily measurable correlates of protection. Furthermore, it has been suggested that antibodies may be more indicative of malaria exposure history in endemic areas rather than representative of a protective response (10).

More complex study designs in malaria endemic areas have consequently been undertaken. These studies involve longitudinal cohorts of children that prospectively document incidence of infection and number of clinical episodes of malaria to determine if naturally acquired immunity to a specific antigen or set of antigens correlates with protection. This type of study design can also address the question of cross-protection where primary infection with one antigenic variant confers protection against infection with a different variant of the same protein. With this in mind, more comprehensive sero-surveys using microarray technology that simultaneously measure a panel of antibody titres to multiple and variant malaria antigens from an extremely small sample volume, have been used to generate a ‘sero-reactivity profile’ for each individual (11). Consistent with classic antibody assays, increasing magnitude and complexity of antibody repertoires were associated with malaria exposure and a subsequent decrease in the incidence of malaria infections (7,11). However, clustering analysis revealed that children with antibodies against apical membrane antigen 1 (AMA-1) and merozoite surface protein 2 (MSP-2) allelic variants, and MSP-1 block 2 were more likely to have asymptomatic infections compared to children with very little reactivity or those who had antibodies against the majority of antigens tested (11). This suggests that antibody repertoires are both quantitatively and qualitatively different in children after the same exposure, and that more antibodies to more antigens are not necessarily more protective. This also supports the theory that ‘blocking’ antibodies that prevent the function of another antibody can develop in some individuals as an immune evasion strategy (12,13) and emphasizes the need for functional antibody assays and thoughtful design of malaria vaccine antigens. By extending the analysis of longitudinal sero-reactivity profiles, the kinetics of the antibody-parasite interaction could be used to determine the rate of parasite clearance and the duration of high titer antibody levels which might provide clues as to the importance of antibody avidity as well as specificity in protection. This concept was explored by Filipe *et al.* (14) using mathematical modelling. These models suggested that clinical immunity developed early in life and was exposure-dependent, while anti-infection immunity developed later in life and was governed by age-dependent biological processes rather than magnitude of exposure. Temporal stability of a panel of anti-malarial antibodies and differential rates of acquisition have yet to be empirically determined in naturally exposed children, but this model is intriguing in that the anti-infection immune profile was dependent on age rather than cumulative exposure. Sero-profiling has given us a more comprehensive picture illustrating the development of an anti-malarial antibody repertoire in young children yet falls short at revealing how a ‘good’ antibody response is induced and maintained and why children do not consistently make one.

Another area that is being given more consideration in malaria vaccine design strategies is IgG subclass switching. An argument can be made for engineering a malaria vaccine to preferentially induce IgG3 antibodies that have been associated with antibody-dependent cellular inhibition of parasite growth (15) and associated with the acquisition of clinical immunity (16). However, little is known about factors that induce class switching and how the relative proportion of IgG1/IgG3 to different malaria antigens is established. Although class switching appears to be influenced by intensity of antigen stimulation and the age of the individual, young children appear to be deficient in promoting IgG3 production (17). There are also reports that even when children produce IgG1 and IgG3 antibodies to merozoite antigens they decay rapidly (18).

The maintenance of serum antibody levels after exposure to antigen, either by infection or immunization, has been referred to as ‘serologic memory’ (19). The mechanisms mediating the development of short-lived antibody secreting cells and long-lived plasma cells are not fully understood. However, it appears that short-term serological memory may be dependent on antigen stimulation whereas long-term serological memory is antigen-independent and depends on homeostatic activation. Several mechanisms involved in plasma cell differentiation and survival have been described, such as B-lymphocyte-induced maturation protein 1 (Blimp1) and A proliferation inducing ligand (APRIL). Blimp1 acts in a concentration-dependent manner to regulate antibody secreting cell differentiation into short- or long-lived plasma cells (20); whereas APRIL, along with other transcription factors, stimulates B-cell proliferation and survival (21). It has yet to be determined if transcriptional control of activated B-cell differentiation and survival is influenced by age or malaria infections. Nonetheless, the notion of short- as opposed to long-lived antibody secreting cells that serve different functions and are preferentially induced early in childhood could explain the counter-intuitive inverse association between age and merozoite inhibition assays observed in two independent studies of naturally exposed children (22,23). Growth-inhibitory assays are an *in vitro* functional means to determine if antibodies found in an individual’s plasma can prevent a merozoite from invading an erythrocyte (22). These assays are malaria specific but do not reveal which anti-malarial antibodies prevent merozoite invasion. In contrast to an overall increase in total IgG antibody titres to most blood stage malaria antigen, the magnitude of *in vitro* growth-inhibitory antibody (GIA) activity decreased with age (children screened were 2–14 years old), even though GIA activity was significantly associated with less risk of malaria infection after adjusting for age (22,23). It is possible that single or few malaria infections may generate low-complexity ‘mono-specific’ short-lived plasma B cells directed against selected antigenic domains of merozoite ligands that are functionally critical to invasion, akin to the situation when malaria-naïve individuals are vaccinated with single candidate antigens (24,25). These B cells may not receive the proper signals to be retained in the long-term memory pool. Studies failing to show protective associations with GIA activity examined children older than 3 years of age and adults from malaria endemic areas (26,27). Thus, these data further support an age-dependent regulation of B-cell differentiation and survival mechanisms to malaria antigens when first experienced early in life.

To determine how much growth-inhibitory activity could be attributed to a single antibody specificity, another *in vitro* functional antibody assay was developed (28). MSP-1 is the most abundant protein on the surface of blood stage Pf merozoites. This protein induces high titre total IgG antibodies against the conserved C-terminal MSP-1 19 kDa (MSP-1_19_) region encoding the B-cell epitopes that are associated with protection against infection and disease (29–31). Given these properties, MSP-1 is being tested as a vaccine candidate (32,33). The *in vitro* MSP-1_19_ invasion inhibitory antibody (MSP-1_19_ IIA) assay utilizes transgenic parasites differing only in their MSP-1_19_ domain and by comparison of activity in parallel cultures can attribute inhibition to antibodies solely against MSP-1_19_ (28). Paradoxical results have been reported for MSP-1_19_ IIA whereby IIA activity was not associated with protection from infection, was very low in asymptomatic malaria-exposed individuals, and did not appear to differ with age (34; Moormann AM, Chelimo K, Dent AE, et al, unpublished data). However, MSP-1_19_ IIA activity was found to be more prevalent during episodes of acute febrile malaria compared to healthy age-matched children from the same malaria holoendemic area (Dent and Moormann, unpublished data); was associated with protection from infection in a highland area where malaria infections are more likely to be symptomatic (35,36); increased during early infancy (6–24 months of age), and was associated with prenatal exposure to malaria (37).

Taken together, a possible interpretation is that MSP-1_19_ generates relatively short-lived antibody secreting cells that are dependent on antigen exposure and may reach levels sufficient to confer clinical immunity if accompanied by fever – which will be explored in more detail within the scope of innate immune priming of adaptive immunity. By comparison, combined anti-malarial antibody activity measured by GIA is composed of longer-lived plasma cells (relative to MSP-1_19_ activity alone) that appear to survive in the absence of antigen and play a role in protection from re-infection. The observation that overall functional anti-malarial antibody activity decreases with age may be countered by the development of more efficient cell-mediated adaptive immunity, in keeping with the concept that different components of ‘protective immunity’ may vary with age.

The role of memory B-cell responses in malaria has recently been reviewed. However, a lack of consensus remains regarding whether the malaria parasite somehow prevents the development and/or maintenance of long-lived plasma cells and memory B cells. Yet there is agreement in that children consistently make poorer responses compared to adults (38). Few studies have directly examined malaria-specific memory B cells in children as these assays require a considerable number of lymphocytes and are more challenging to carry out. One such study concluded that a significant proportion of antibodies to AMA-1 and cysteine-rich interdomain region 1α (CIDR1α), a parasite-derived erythrocyte membrane protein, existed in the absence of cognate memory B cells. In contrast, MSP-1_19_ specific memory B cells were not producing antibodies in asymptomatic individuals (39). The authors speculated that MSP-1_19_ specific memory B cells may be stimulated to produce anitbodies upon reinfection. However, another group went on to further characterize memory CD19^+^ B cells in malaria-infected children from Mali and found a significant proportion with an ‘exhausted’ phenotype (Weiss and Crompton, personal communication) compared to individuals from nonendemic areas. A general definition of an exhausted lymphocyte is one that is antigen-experienced, has undergone some differentiation but is no longer functional. This subset of tissue-homing dysfunctional memory B cells (CD20^hi^/CD27^lo^, C21^lo^) found in malaria-experienced children expressed an inhibitory receptor Fc-receptor-like-4 similar to those described in HIV-infected individuals who undergo persistent antigen stimulation (40). Poor antibody responses may therefore be the results of prematurely exhausted B cells. Another study examining B-cell subsets in malaria-exposed Kenyan children provides additional evidence that malaria infections influence the rate of B-cell maturation and homeostasis. During acute malaria infections, there is an overall decrease in total CD19^+^ peripheral B-cell populations, yet further delineation of B-cell subsets demonstrates a transient increase in the proportion of CD38^+^ IgD-memory B cells (41,42). Lymphopenia and hypergammaglobulinaemia have long been recognized as complications of acute malaria in children (43,44), yet how malaria impacts the B-cell compartment is not well understood. Through direct interaction with memory B cells via the CIDR1α of the Pf erythrocyte membrane protein 1 (45,46), malaria could be driving memory cells toward plasma cell differentiation and migration back to the bone marrow. Thus, the concern that parasite-derived mediators could adversely influence the maintenance of long-lived humoral immunity to malaria vaccines in chronically infected populations may be well founded, with children being particularly susceptible.

Finally, the mechanisms by which anti-malarial antibodies are generated by a vaccine or natural infection are being considered, especially in paediatric populations. Age-dependent differences have been described for T-cell independent (TID) B-cell antibody responses and T-cell dependent (TD) antibody responses [reviewed in (47)]. During the first year of life, neonates are deficient in TID B-cell responses and tend to have less robust TD antibody responses in the absence of repeat stimulation [review in (48)]. As IFN-γ producing CD4^+^ T cells have been implicated in protective immunity to both liver and blood stage infections, an efficacious vaccine for infants and young children aims to not only induce CD4^+^ T cell help but ‘boost’ the level of TID antibody production above that which occurs during a natural malaria infection to levels observed for adults.

## Cell-Mediated Immunity

Cell-mediated adaptive immunity is arguably as important in protection against malaria as humoral immunity; the discovery and details of which have been extensively reviewed by others (8,49,50). Suffice it to say, a pro-inflammatory response is desirable to clear parasites but when this response in not appropriately regulated, immunopathology ensues (3). The landmark prospective study of Javanese transmigrants confirmed that adults develop semi-protective immunity faster than children even though adults suffered more severe disease compared to children during initial malaria episodes (51). This study highlights the understanding that cumulative exposure alone is not sufficient for developing semi-protective immunity against malaria, leading us to examine intrinsic reasons why young children’s immune responses render them susceptible to malaria. In addition, this study illustrates the age-associated difference in immunopathology with children appearing to be hyporesponsive upon primary infection. A distinction between immunity that prevents malaria infections and that which prevents disease continues to resonate. As severe malaria is associated with sustained and exaggerated pro-inflammatory responses, the onus falls on a better understanding of down-regulatory mechanisms that may be insufficiently modulated early in life.

Measuring the network of cell-mediated immune responses has additional challenges over assays designed to measure humoral immunity, including ethically justified limitations in blood sample volume, especially from children under 5 years of age. Therefore, such studies are sorely lacking in human malaria vaccine trials, especially those conducted in children. However, recent advances in multiplex technologies have begun to shed light on the full functional potential of antigen-specific T-cell responses against many infections, including malaria. Luminescent microsphere assays and multiparameter flow cytometry have significantly expanded our ability to examine human lymphocyte populations and their functions. Resources availed for capacity building and technology transfer in malaria endemic countries have also facilitated more sophisticated investigations of human cell-mediated immunity.

A plethora of cytokine profiles have subsequently been revealed, similar to sero-profiles, that give us a broader picture of cell-to-cell signalling in response to a malaria infection. This technology has been used to investigate the kinetics of cytokine responses in a sporozoite challenge study in malaria-naïve adults (52). Walther *et al.* reported considerable variation between individuals during the course of their malaria infection and thus categorized them into three groups: those with moderate levels of IFN-γ and IL-10, but no IL-12p70; those with IL-12p70 and high levels of IFN-γ and IL-10; and those that failed to up-regulate pro-inflammatory cytokines but had the highest TGF-β levels in response to Pf-infected erythrocytes *in vitro*. Pro-inflammatory *in vitro* recall responses (i.e. IFN-γ, IL-12p70, TNF-α and the ratio of TNF-α to bioactive TGF-β) were associated with the ability to control parasite growth (length of time to first detection of 1000 parasites/mL) after challenge infection. In contrast, higher ratios of IL-12 or TNF-α to IL-10 were correlated with a more rapid onset of clinical symptoms (i.e. fever, headache and malaise). This study clearly demonstrates individual host differences in the magnitude and quality of cell-mediated immunity despite the same parasite exposure, and hints at the far-reaching consequences of innate immune signals in generating protective adaptive immunity. Simultaneously measuring the sequence and magnitude of pro-inflammatory events (i.e. IFN-γ, TNF-α and IL-6) in conjunction with antigen presentation signals (i.e. IL-12 and IL-18), balanced by down-regulatory cytokines (i.e. IL-10, TGF-β) within the context of a malaria infection or vaccine trials in children would significantly improve our understanding of immunologic harmonization (or lack thereof) to specific malaria antigens. Especially if these children continue to be exposed to natural infections that may subvert the desired response. Longitudinal repeat measurements of relative cytokine contributions during the course of a malaria infection also gives us the ability to determine which down-regulatory mediators are missing, suboptimal or delayed, and may, by their absence, contribute to the development of immunopathology, especially in paediatric populations.

Taking the lead in utilizing and optimizing polychromatic flow technology are studies of acute and persistent viral infections which describe a mosaic of T-cell subsets associated with immune memory and protection [reviewed in (53)]. Multiparameter (at least six colours) flow studies have described four important findings about the quality and magnitude of T-cell responses: (1) antigen concentration and duration of T-cell stimulation influence phenotypic heterogeneity; (2) multi-functional (i.e. more than one cytokine being secreted from the same cell) T cells express more IFN-γ per cell than mono-functional T cells; (3) multi-functional T cells are more likely to differentiate into long-term memory cells and (4) protective antigen-specific T-cell receptor repertoires display shared, or so-called public, specificities; i.e. dominance of particular clonotypes that recognize the same epitope and are found in a majority of individuals (53,54). What is not yet known are which factors, including age, regulate the induction and maintenance of T cells displaying the distinct phenotypes associated with protection from severe disease. It would appear that finite exposure to antigen such as those seen during acute infection tends to generate long-lasting memory T cells whereas chronic, persistent infections induce T-cell exhaustion, anergy or deletion via apoptosis (55). For children living in endemic areas, malaria can be an acute or chronic infection with high- or low-density parasitemia depending on intensity of malaria transmission, degree of maternal antibody protection, prenatal exposure, and access to prompt diagnosis and treatment [reviewed in (56)]. Taking into consideration these variables, one could then speculate that a child with a high-density malaria infection may generate many immediate, short-lived mono-functional effector T cells expressing only IFN-γ yet not differentiating into long-term memory cells, leaving the child susceptible to repeat infections. Models for signal-strength dependent T-cell differentiation support this notion, suggesting that T-cell stimulation has to be ‘just right’ in order to induce T-cell memory (57–59). Tangentially, differences in IFN-γ intensity from mono- compared to tri-functional T cells may explain the discordance observed between enzyme-linked immunosorbent spot (ELISPOT) and ELISA assay results to the same antigen and why the cytokine level measured by ELISA correlates with protection from infection when the frequency of IFN-γ producing cells specific to MSP-1 does not (Moormann AM, Chelimo K, Dent AE, et al, unpublished data). If, on the other hand, an individual from an epidemic-prone region becomes infected after only a few infectious mosquito bites, this person’s T-cell response might be similar in quality to an acute viral infection and generate long-term memory cells. Evidence supporting this possibility comes from immunologic studies of individuals from highland areas, where the incidence and prevalence of malaria infections are extremely low for prolonged periods of time but who are still able to generate malaria-specific IFN-γ recall responses to pre-erythrocytic and erythrocytic stage malaria antigens (60,61). The importance of identifying antigen-specific T-cell memory phenotypes (i.e. using memory and differentiation markers) and effector functions (i.e. cytokine and granzymes/perforin) in response to malaria has begun to be explored in malaria-naïve volunteers participating in phase I trials of MSP-1 (62) and in animal models of circumsporozoite surface protein (CSP), the leading pre-erythrocytic stage malaria vaccine candidate (53).

Compounding the issue of T-cell naïveté is evidence that neonates are biased *in utero* against a strong pro-inflammatory response as a residual effect of having recently been in the intra-uterine environment [reviewed in (63)]. This state renders neonates and young children at risk for infection and impairs their responses to many vaccines [reviewed in (64)]. Because of the naturally delayed production of T_H_1 mediated pro-inflammatory cytokines (i.e. IL-12 and IFN-γ), neonatal immunity is thought to be ‘suppressed’. However, more recent studies have shown that neonatal monocytes and antigen-presenting cells do express pro-inflammatory and regulatory cytokines (i.e. IL-6, IL-10 and IL-23) and at higher levels than adults (65–67). In addition, a third, independently differentiated, arm of the adaptive T-cell immune response has recently been described, T_H_17 cells [reviewed in (68)]. T_H_17 cells appear to be an early, sequential response to pathogens prior to the induction of a T_H_1 or T_H_2 response (69). In humans, naïve CD4^+^ T cells, after stimulation with IL-1β, IL-6 elicited from activated monocytes via innate immune pathways (70), secrete IL-17 and induce pro-inflammatory cytokines (i.e. IL-6, IL-1β and TNF-α) and chemokines (i.e. IL-8) increasing neutrophil recruitment to clear extracellular pathogens. T_H_17 cells are also stimulated with IL-21 and IL-22 in an autocrine and paracrine fashion by NK cells (68). Curiously, IL-22 increases acute-phase reactants in hepatocytes (71) and protects them against acute liver inflammation (72). The influence of TGF-β on T_H_17 cell induction may differ in mice and humans but appears to divert activated T cells away from T_H_1, T_H_2 and T_H_17 cell differentiation and toward the induction of FOXP3+ regulatory T cells (iTreg). Mouse models have been used to examine these four T cell subsets during a *P. yoelii* malaria infection and found that IL-10 producing Tregs impede parasite clearance (73). Taken together, immune equilibrium could be mediated between T_H_17 – Tregs early in life until natural ‘suppressors’ of T_H_1 immunity have been lifted as an infant ages. Focusing on the few studies of children, those with a higher incidence of malaria had higher frequencies of CD4+ CD25^hi^ regulatory T cells that prevented the protective IFN-γ responses characteristically observed in adults who have developed anti-malarial immunity (74). Without longitudinal repeat measurements it is unclear if a pro-inflammatory response was at one time induced in these children or if the down-regulatory response was over zealous or abnormally sustained and prevented induction of an IFN-γ response. Cord blood studies, used as surrogates to examine neonatal lymphocytes, have demonstrated that Tregs are particularly abundant at birth (75), providing additional evidence that down-regulatory mechanism may be normally more pronounced during early infancy.

Another area that has received considerable attention to explain how chronic viral infections can persist is the phenomena of T-cell exhaustion, whereby T cells acquire antigen-specific effector function but gradually become less functional as the infection progresses. There appears to be a complex layer of synergist, nonredundant inhibitory receptors involved in the regulation of T-cell exhaustion, such as programmed death 1 and lymphocyte activation gene-3, that correlate with severity of infection (76). Preliminary age structured cross-sectional studies comparing expression levels of these inhibitory receptors on human T cells show higher levels of expression in children that are inversely correlated with age (JE Wherry, personal communication). This provides a possible mechanism by which infants and young children normally ‘suppress’ pro-inflammatory T-cell function that could otherwise induce immunopathology. These inhibitory receptors have also been implicated in regulating T-cell differentiation into memory (77,78). However, it is unknown whether the malaria parasite influences the expression and function of these inhibitory receptors. It is possible that children who experience severe manifestations of malaria are not able to properly regulate these inhibitory pathways compared to children with similar malaria exposures, but milder symptoms. Comparing the immune responses of children with mild reactions who are able to control the infection to the responses of children with severe reactions may offer clues as to whether inhibitory pathway function is an essential component of the pediatric immune response to malaria infection.

Overall, cell-mediated immunity involves the induction and proliferation of antigen-specific T cells that will clear the infection by direct cytotoxic killing or by assisting with the formation of antibodies to mediate parasite clearance. This is then followed by a contraction phase whereby certain antigen-specific T cells survive as memory – or not. Little is known about what regulates the contraction and differentiation into memory phases of a normal T-cell response let alone how this process may falter in infants or how malaria may prevent memory T-cell differentiation and survival. Important insight into these down-stream events may lie in the nuances of the innate immune signature from malaria.

## A Pivotal Role for Innate Immunity

Escalating interest in how innate immune cells and signals initiate the quality of adaptive immunity has increased our understanding of immune responses to malaria [reviewed in 79,80)]. Innate immune lymphocytes capable of recognizing a pathogen without prior stimulation include DCs, monocytes, macrophages, NK cells, NK T cells and gamma-delta (γδ) T cells. When DCs or monocytes-macrophages encounter a pathogen and release cytokines such as IL-12, IL-15, IL-18, TNF-α and IFN-α/β, NK cell are activated; whereas IL-4, IL-10 and TGF-β suppress NK cell function. During malaria infections, and in response to IL-12, NK cells are the first to secrete IFN-γ (with γδ T cells and NK T cells responding later) and are able to directly kill Pf-infected erythrocytes (81). Individual differences in NK responsiveness may lie in the underlying human genome, such as functional allelic polymorphisms in killer cell inhibitory receptors and toll-like receptors (TLR) (82,83). Yet, embracing the complexity that constitutes the human immune milieu, the recognition of IL-12 and IL-23 in orchestrating the development of T_H_1 and T_H_17 subsets, respectively, pose new questions in T-cell decision making [reviewed in (84)]. IL-12 and IL-23 are two heterodimeric cytokines that share the p40 chain to become biologically active. IL-12 and IL-23 are both inhibited by IL-10, however DCs induced to differentiate in the presence of prostaglandin E_2_ (PGE_2_) produce more IL-23 than IL-12 (85). Interestingly, PGE_2_ has been shown to inhibit INF-γ production from naïve human cord blood mononuclear cells (CBMCs) (86) and TD Ig production by neonatal lymphocytes (87). PGE_2_ and IL-10 have been implicated as mediators that bias foetal and neonatal immunity away from excessive IFN-γ production that would be deleterious at the maternal–foetal interface (88). The role for the IL-23/IL-17 axis in response to malaria during infancy has yet to be explored.

The few studies directly investigating the impact of malaria on human DC function have yielded conflicting results. A recent balanced review attempts to resolves these apparent contradictions concluding that disparate findings could be explained by low-dose immune induction vs. high-dose immune suppression (89). The authors suggest the dose-dependent DC response sequentially varies during the course of an individual infection from early, low-dose responsiveness to late, high-dose nonresponsiveness. Dose-dependent DC responsiveness could be extended to nonimmune infants, after prolonged periods of high malaria parasitemia, compared to semi-immune adults who are able to restrict infections to lower densities. However, this model does not take into account that DC function is fundamentally impaired in neonates and young children [reviewed in (64)] and that an increasing body of research implicates impaired responses to TLR ligands [reviewed in (88,90)] in poor infant immunity to infectious diseases.

As priming the adaptive immune system appears to be essential to inducing the desired response to a vaccine, innate immune signatures and adjuvants are deservedly earning more attention. Innate immune cells are activated via TLRs that express pattern-recognition receptors used to identify pathogen-associated molecular patterns. The list of TLR ligands now includes malaria-derived molecules: Pf toxin, glycosylphosphatidylinisitol is recognized by TLR2 and TLR4, whereas parasite DNA complexed with haemozoin is a TLR9 ligand (91–93). Interestingly, TLR2 appears to be the key that preferentially induces IL-23 expression in lieu of IL-12 (94). Recent studies have evaluated TLR expression and responsiveness in humans infected with malaria. Adults with severe and mild malaria had increased expression of TLR2 and TLR4 on CD14^+^ monocytes and myeloid DCs and decreased expression of TLR9 on plasmacytoid DCs compared to adults with no history of malaria exposure (95). Malaria-naïve adults experimentally infected with Pf had increased expression of TNF-α, IL-1β and IL-6 in response to a TLR4 ligand (LPS) and IL-6 and IL-10 in response to TLR2 + TLR1 ligand (Pam3Cys) on day 8 of the infection (96). *In vitro* peripheral blood mononuclear cell (PBMC) pre-incubation experiments with Pf followed by TLR ligand stimulation demonstrated a temporal-synergistic activation of TLR-4 and TLR-2 significantly increasing TNF-α and IFN-γ but reducing IL-10 production. This is a distinct contrast to the effect observed when PMBCs are primed with LPS, which induces subsequent TLR tolerance (97). Similarly, naturally Pf-infected children had increased TLR2 expression on CD14^+^ monocytes isolated from PBMCs compared to uninfected children (aged 5–16 years old) and enhanced TLR2 (Pam3Cys) activation but in this case resulted in higher production of both IL-10 and TNF-α (98). Consistent with a Pf-enhanced TLR responsiveness, naturally ‘primed’ (*in utero* exposure) CBMCs produced more IFN-γ in response to both TLR3 (poly I : C) and TLR4 (LPS) ligand stimulation if the mother was infected with malaria during the last month of pregnancy, whereas CBMCs without prenatal malaria exposure had significantly higher TNF-α in response to the same ligands (99). In this study, no differences were observed for IL-10 production stimulated with TLR3 or TLR4 ligands and maternal malaria history. This supports other neonatal studies demonstrating differential effects of *in utero* Pf exposure on adaptive immune responses to malaria (100). Analogous to studies of adaptive immunity, seemingly inconsistent results for innate immune responses appear when temporal, spatial and epidemiologic elements such as age and co-infections are not taken into account. Clearly more studies are needed to fully appreciate the synergy or antagonism of innate immune signals in children compared to adults, and the impact of co-infections, when evaluating malaria vaccine efficacy.

## Malaria Vaccines Efficacy in Children thus Far

For ethical reasons, malaria vaccines are tested for safety, immunogenicity and efficacy in age de-escalating clinical trials prior to reaching the ultimate target population: infants <1 year of age. Therefore, deciding which vaccines are eventually tested in young children is determined by results generated from adult trials. Many malaria vaccine candidates and adjuvant combinations have been tested in phase I and II trials of adults with fewer reaching phase III trials in children and fewer still with encouraging results [reviewed in (101–103)]. Here, we describe two promising vaccine trials conducted in young children: a pre-erythrocytic stage vaccine candidate, RTS,S that targets the CSP combined with an adjuvant system AS01E; and a multi-valent blood stage formulation, combination B comprised of MSP-1, MSP-2, and ring-infected erythrocyte surface antigen (RESA) and the adjuvant Montanide ISA 720.

The goal of RTS,S is to generate neutralizing humoral immunity directed against the sporozoite stage of the malaria parasite before they invade the liver, in addition to inducing CSP-specific CD8 and CD4 Th1+ T cell immunity. An RTS,S/AS01E efficacy trial was conducted in Kenya and Tanzania from March through August 2007 in which children 5–17 months of age were randomly assigned to receive either the malaria vaccine (*n* = 402) or the control rabies vaccine (*n* = 407). The cumulative 8-month incidence of all clinical malaria episodes was 9% in the group that received RTS,S compared to 21% in the control group, with an adjusted efficacy of 56% (95% CI: 31–72; *P* < 0.001) (104). Vaccine immunogenicity was determined by measuring titres of anti-CSP antibodies by standard enzyme-linked immunosorbent assay units (EU). The majority of children in both arms of the study had detectable low level anti-CSP antibody titres however the children receiving RTS,S had significantly higher titres 3 months post-immunization compared to the rabies vaccine control group. These antibodies titres waned over the subsequent months of follow-up, and there was no evidence that higher titres were associated with greater protection against clinical disease than lower titres. The efficacy of RTS,S/AS01E was similar to the previous year’s study with the AS02D adjuvant formulation in 2-month-old Tanzanian infants at 65.2% (95% CI: 20–84.7; *P* = 0.01) (105). Of note the geometric mean anti-CSP titre in the 5-month-old children 3 months after the first dose of RTS,S/AS02D was 69.5 EU/mL compared to the slightly older children (5–17 months old) who had anti-CSP antibody titres of 539.6 EU/mL 3 months after first dose of RTS,S/AS01E. These antibody titres were fivefold lower than baseline measurements in semi-immune adults (106,107). The reason for improved antibody titres in the older children may have been due to the immunogenic superiority of AS01 compared to AS02 adjuvant formulations; however, age of administration cannot be ruled out since direct comparisons between different adjuvants do not necessarily correspond to differences in antibody titres (108). Choice of adjuvant can nonetheless significantly impact strength and duration of IFN-γ and IL-5 responses to RTS,S (108) that may fight malaria independent of antibodies. Assessments of cell-mediated immunity were not reported for these trials in children, yet could have provided additional clues as to how RTS,S mediates protection and why higher antibody titres were not predictive. Combination B, composed of malaria blood stage proteins to MSP-1, MPS-2 and RESA aimed to generate humoral as well as IFN-γ mediated immunity. This vaccine was tested in children 5–9 years old living in Papua New Guinea and included measurements of antibody titres and cytokines levels (i.e. IFN-γ, TNF-α, IL-4 and IL-10) (109). This study also assessed the influence of clearing pre-existing malaria infections on the development of immunity and controlling parasite densities. Genton *et al.* found that antibody levels to all three antigens were increased by the vaccine but only MSP-1 induced IFN-γ recall responses (which may have been due to how this vaccine was constructed as it was fused with a universal T-cell epitope derived from CSP). The vaccine did not induce IL-10 or TNF-α however IL-4 levels were lower in response to MSP-1 though not to MSP-2 or RESA. Clearing circulating malaria parasites prior to immunization appeared deleterious for inducing MSP1-specific IFN-γ responses; perhaps by preventing ‘boosting’ afforded by natural infections. This vaccine reduced parasite density (only in those not pretreated for malaria infection) but did not reduce prevalence of infection and was strain-specific for MSP-2. Similar to RTS,S, immunizing children with combination B failed to achieve anti-malarial antibody levels observed in adults and titres significantly waned over time.

## Malaria-Exposure Reduction Programmes

As malaria vaccine trials in infants provide optimism, progress has been made in decreasing the incidence of clinical and severe malaria in young children as the result of exposure-reduction programmes such as insecticide-treated bednets (ITBN) and intermittent preventative therapy in infants (IPTi). Both of these interventions decrease the cumulative parasite burden by preventing some (but not all) malaria infections, with the latter also decreasing the duration of asymptomatic infections experienced by a child (110). It is important to note that these interventions are malaria-disease prevention measures targeting young children and pregnant women, with most of the children typically in good health and not experiencing symptoms of malaria – and thus good candidates to participate in malaria vaccine studies.

Monitoring the impact of these strategies on decreasing the incidence of clinical and severe malaria has yielded some interesting findings that are relevant to understanding the development of anti-malarial immunity in infants and young children. Several studies have addressed the possibility that exposure-reduction programmes could result in a delay in acquisition of immunity thereby shifting clinical sequelae to an older age group – referred to as a rebound effect. Evidence from ITBN studies has alleviated this concern as bednets appear to mediate reductions in malaria-associated morbidity by decreasing parasite densities and delaying median age of first parasitaemia from 4 to 10 months of age (111). Even though ITBN coverage reduced IgG antibody responses to pre-erythrocytic antigens, there was no apparent difference in IgG antibody levels to blood stage antigens in children <2 years of age (112). Equally, studies examining the impact of varying IPTi schedules on the development of clinical immunity demonstrated a decrease in the incidence of severe malaria during the first year of life and a delay in time to first infection on an average of 2 months (113). Decreases in clinical malaria were consistently observed regardless of anti-malarial prophylaxis frequency (e.g. weekly compared to three times within the first 12 months of life) (113–116). However, there was an increase in the incidence of severe malaria observed during the second year of life in children who received continuous, weekly chemoprophylaxis during their first year of life compared to the placebo group (115,117). The authors concluded that malarial anaemia was a function of the age at which infection takes place (i.e. <1 year of age) lending support to the hypothesis that intrinsic pathophysiological mechanism are involved in risk of severe malaria. This observation could also implicate maternal antibody protection as being important for the development of infant immunity by providing a ‘buffer’ during an infant’s first exposure to malaria and thereby attenuating pathologic antigen stimulation. This clinical rebound effect was no longer apparent once the children reached 4 years of age and has been abrogated by following a less intense prophylactic treatment schedule (i.e. three times, at 3, 4 and 9 months of age in conjunction with the Expanded Program on Immunization) (114).

A parallel assessment of humoral immune responses within the context of a three-dose IPTi schedule was conducted in the study by Quelhas *et al.* (113). Antibody levels in those who were given IPTi did not significantly differ compared to placebo controls, suggesting that IPTi did not delay acquisition of humoral immunity. However, differences were observed for those given IPTi who also had at least one episode of clinical malaria (i.e. fever) compared to those experiencing clinical malaria without chemoprophylaxis. For children experiencing a febrile malaria episode, those on ITPi generated higher antibody levels for MSP-1_19_ (3D7) and AMA-1 (3D7) compared to placebos starting at 9 months of age, the point at which maternal antibodies are waning and infant immunity develops (113). This scenario hints at two possible mechanisms. First, a moderate or limited antigen dose, resulting from the prophylactic anti-malarial administration, may be more permissive for inducing a robust antibody titre compared to a higher antigen dose. Second, children given IPTi resulting in less chronic asymptomatic malaria infections were more likely to generate higher antibody titres when they experienced a febrile malaria episode, supporting a pivotal role for innate immune mediators in priming anti-malarial immunity. These studies support the notion that in addition to extrinsic factors that may modulate the dose and duration of the infection there is an age-dependent physiologic component involved in the development and maintenance of functional immunity that is not well understood. Interplay between exposure-reduction interventions and the development and maintenance of protective immunity to malaria in children is just beginning to be explored. Thus, a child’s participation in an integrated malaria prevention program may actually improve the development of anti-malarial immunity.

## A Way Forward

The most evident impediment against basic immunologic investigations of infants and young children are limited blood volumes, an acceptable frequency of repeat sample collections, and gaining ethical and parental approval for enrolling often healthy children in research studies. Unfortunately, these understandable obstacles have left children, who are the most vulnerable to the adverse consequences of malaria, understudied. In the midst of technological advancements and ethical deliberations, some of these long-standing issues are being overcome. Yet, if a malaria vaccine is deemed efficacious in adults the question remains if that vaccine will also protect infants and young children. Current opinion in the field of immunology is that the next generation of vaccines will need to be designed to do what nature does not in order to confer long-lasting protective immunity. With the ultimate goal of designing a malaria vaccine that will be delivered within the first year of life, when there appear to be inherent down-regulatory mechanisms preventing the induction and maintenance of protective immunologic memory, the composition of such a vaccine may need to differ from one that would protect an adult. It may not be enough to teach an infant immune system to behave like an adult’s by priming and boosting a pro-inflammatory response against an array of malaria antigens that in turn induce potent humoral immunity. A malaria vaccine for infants may also need to be designed to subsequently mute pro-inflammatory responses and further guide the selection of the appropriate T and B cells to be maintained as memory – a ‘prime-boost-mute-select’ vaccine design, if you will. Therefore, increasing our understanding of basic immunologic differences between adults and young children will provide the mechanistic insights necessary to design a malaria vaccine that will be safe and successful in infants.
